# How to Cure Rhus Poisoning

**Published:** 1894-01

**Authors:** 


### HOW TO CURE RHUS POISONING.

Editor of The Homœopathic Physician.

Sir:—Will you kindly allow me space to say a few words upon the treatment of Rhus poisoning, which was noticed in your August number, at page 445, and in the October number at page 509. In the vesicular stage it readily yields to two or three doses of Sanguinaria-canadensis$^{200}$ (B. & T.). When it has been neglected, and has become pustular, Croton-tig.$^{500}$ (B. T.) has never failed to cure. I have used this treatment for twelve years, and have found it invariably successful.

A Layman.

